# Synthesis of Pyrimidine Conjugates with 4-(6-Amino-hexanoyl)-7,8-difluoro-3,4-dihydro-3-methyl-2*H*-[1,4]benzoxazine and Evaluation of Their Antiviral Activity

**DOI:** 10.3390/molecules27134236

**Published:** 2022-06-30

**Authors:** Victor P. Krasnov, Vera V. Musiyak, Galina L. Levit, Dmitry A. Gruzdev, Valeriya L. Andronova, Georgii A. Galegov, Iana R. Orshanskaya, Ekaterina O. Sinegubova, Vladimir V. Zarubaev, Valery N. Charushin

**Affiliations:** 1 Postovsky Institute of Organic Synthesis, Russian Academy of Sciences (Ural Branch), Ekaterinburg 620108, Russia; vvmusiyak@ios.uran.ru (V.V.M.); ca512@ios.uran.ru (G.L.L.); gruzdev-da@ios.uran.ru (D.A.G.); charushin@ios.uran.ru (V.N.C.); 2 Institute of Chemical Engineering, Ural Federal University, Ekaterinburg 620002, Russia; 3 Ivanovsky Institute of Virology, Gamaleya National Research Center of Epidemiology and Microbiology, Ministry of Health of the Russian Federation, Moscow 123098, Russia; andronova.vl@yandex.ru (V.L.A.); g.galegov@yandex.ru (G.A.G.); 4 Saint Petersburg Pasteur Research Institute of Epidemiology and Microbiology, Saint Petersburg 197101, Russia; deina89@mail.ru (I.R.O.); sinek489@gmail.com (E.O.S.); zarubaev@gmail.com (V.V.Z.)

**Keywords:** pyrimidine, purine, 7,8-difluoro-3,4-dihydro-3-methyl-2*H*-[1,4]benzoxazine, antiviral activity, HSV-1, influenza virus

## Abstract

A series of pyrimidine conjugates containing a fragment of racemic 7,8-difluoro-3,4-dihydro-3-methyl-2*H*-[1,4]benzoxazine and its (*S*)-enantiomer attached via a 6-aminohexanoyl fragment were synthesized by the reaction of nucleophilic substitution of chlorine in various chloropyrimidines. The structures of the synthesized compounds were confirmed by ^1^H, ^19^F, and ^13^C NMR spectral data. Enantiomeric purity of optically active derivatives was confirmed by chiral HPLC. Antiviral evaluation of the synthesized compounds has shown that the replacement of purine with a pyrimidine fragment leads to a decrease in the anti-herpesvirus activity compared to the lead compound, purine conjugate. The studied compounds did not exhibit significant activity against influenza A (H1N1) virus.

## 1. Introduction

**Figure 1:**
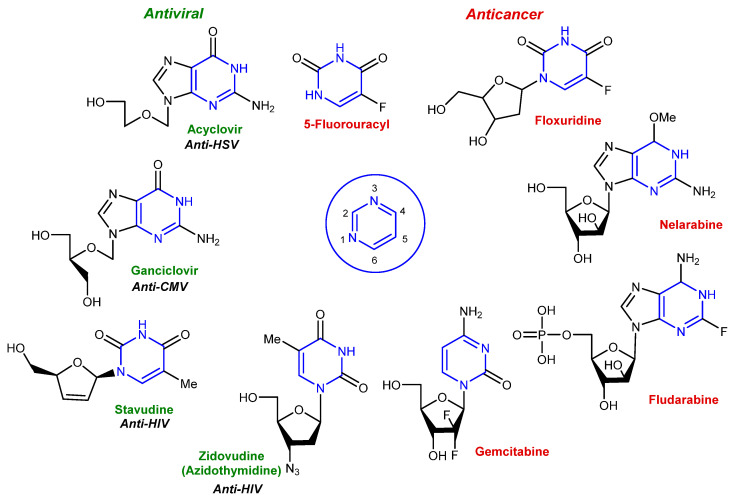
Structures of selected pyrimidine-based antiviral and anticancer agents.

Pyrimidine (1,3-diazine) and its fused derivative, purine (imidazo[4,5-*d*]pyrimidine), are the most important class of heterocyclic scaffolds that form pyrimidine (uracil, thymine, and cytosine) and purine (adenine and guanine) nucleobases, which are structural fragments of nucleic acids. Pyrimidine-based compounds exhibit a wide range of biological and pharmacological activities, including anticancer, anxiolytic, antioxidant, antiviral, antifungal, anticonvulsant, antidepressant, antibacterial, etc. (for some recent reviews, see [1,2,3,4,5,6] and references therein). Derivatives of pyrimidine and purine, as well as nucleosides based on them, are part of efficient medicinal agents (Figure 1) used in the treatment of a wide range of diseases, primarily cancer and viral infections.

The mechanism of action of antiviral agents is primarily determined by selective blocking of metabolic pathways in which nucleobases are actively involved. Recently, researchers have paid great attention to the search for new efficient antiviral agents among pyrimidine [7,8,9,10,11,12,13,14,15,16] and purine [17,18,19,20,21] derivatives, which is especially important in connection with the need to create treatments for SARS-CoV2 (COVID-19) coronavirus infection.

**Figure 2:**
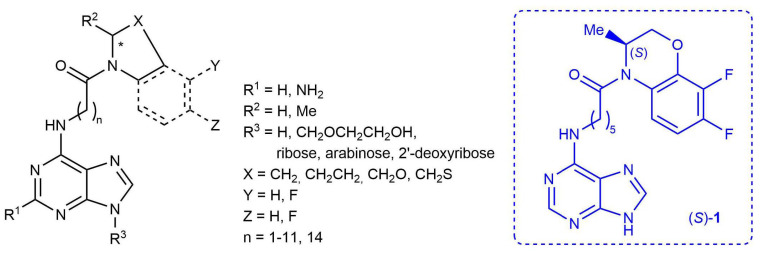
Structures of the previously obtained purine conjugates with *N*-heterocycles.

In recent years, our efforts have been focused on the search for efficient antiviral agents among purine and 2-aminopurine derivatives. For this purpose, we synthesized a number of conjugates of purine and 2-aminopurine with various *N*-heterocycles, including chiral ones, which are attached via a linker, the omega-aminocarboxylic acid NH(CH_2_)_n_CO residue, to position 6 of the purine nucleus (Figure 2) [22,23,24,25,26,27,28,29,30,31]. During our investigations, we varied the structure of the heterocycle (including the configuration of the chiral center in the case of chiral compounds), the length of the polymethylene chain of the linker, and the substituent at the N^9^ atom of purine. Most of the resulting compounds were tested against herpes simplex virus type 1 (HSV-1), including the acyclovir-resistant strain. Compounds containing a linker fragment with *n* ≥ 5 are of the greatest interest in terms of anti-herpesvirus activity. The most active compound turned out to be a purine conjugate with (*S*)-7,8-difluoro-3,4-dihydro-3-methyl-2*H*-[1,4]benzoxazine attached via a 6-aminohexanoyl residue (*n* = 5, compound (*S*)-**1**) [27,28,29]. We have demonstrated that it is the combination of difluorobenzoxazine and purine fragments in one molecule that leads to high anti-herpesvirus activity, including that against the acyclovir-resistant HSV-1 strain [27].

In this work, in order to elucidate the role of the purine fragment in the antiviral activity exhibited by the lead compound, we synthesized a series of derivatives, in which the purine fragment was replaced by a pyrimidine one, and studied the antiviral activity of the synthesized compounds against HSV-1 and influenza A (H1N1) virus.

## 2. Results and Discussion

### 2.1. Chemistry

**Scheme 1:**
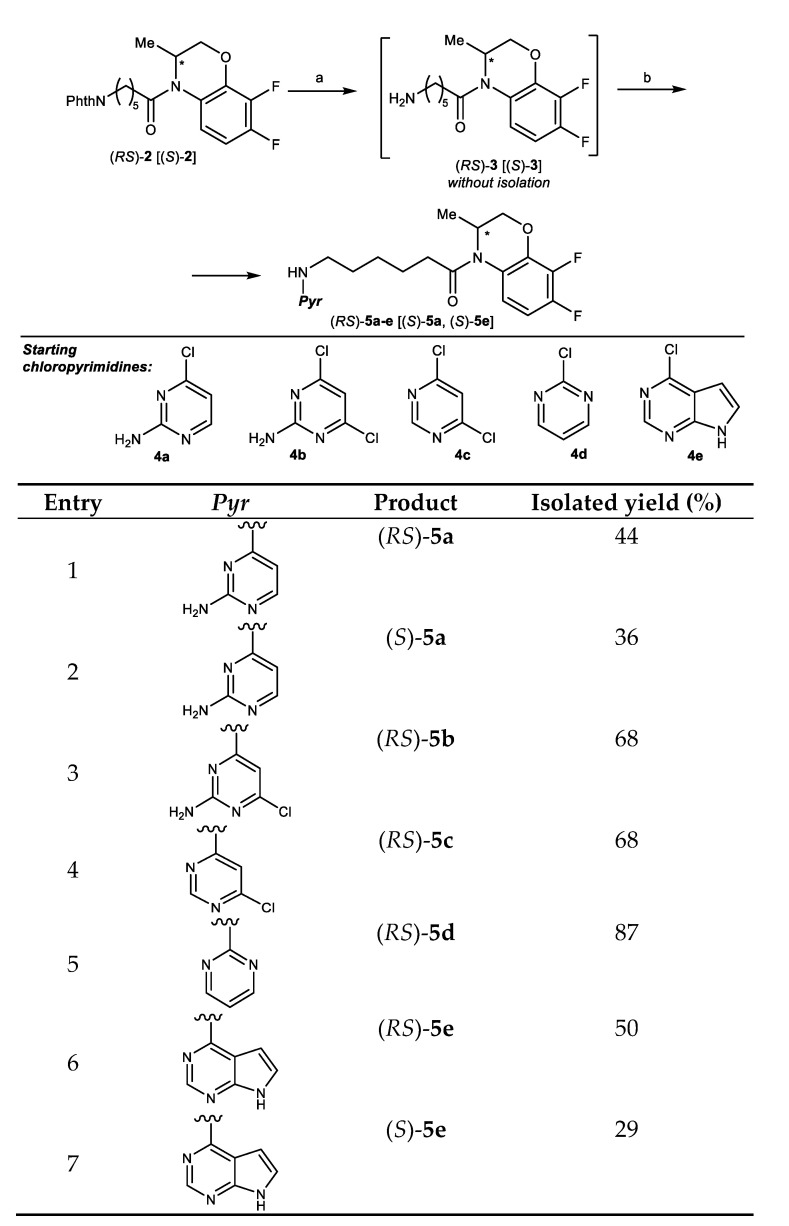
Synthesis of the target compounds **5a**–**e**. (**a**) NH_2_NH_2_ × H_2_O, EtOH, reflux, 2 h; (**b**) **4a**–**e**, Et_3_N, *n*BuOH, reflux, 8 h.

To obtain the target pyrimidine conjugates, we used a synthetic sequence developed earlier for the preparation of purine and 2-aminopurine conjugates [27,30,31]. The starting compounds were amides of *N*-phthalimidohexanoic acid and racemic 3-methyldifluorobenzoxazine ((*RS*)-**2**) and its (*S*)-enantiomer ((*S*)-**2**) [23] (Scheme 1). The removal of the phthaloyl protecting group was carried out according to the standard method of hydrazinolysis in boiling ethanol; the resulting compounds (*RS*)-**3** or (*S*)-**3** with a free amino group were introduced into the reaction of nucleophilic substitution of chlorine in the corresponding chloropyrimidines: 2-amino-4-chloropyrimidine (**4a**), 2-amino-4,6-dichloropyrimidine (**4b**), 4,6-dichloropyrimidine (**4c**), 2-chloropyrimidine (**4d**), and 4-chloro-7*H*-pyrrolo[2,3-*d*]pyrimidine (or 7-deazapurine, **4e**). 

The reaction of nucleophilic substitution of chlorine in chloropyrimidines was carried out under reflux in *n*BuOH in the presence of a tertiary amine, triethylamine (Et_3_N), as an HCl acceptor (Scheme 1). The reaction products were purified by silica gel flash chromatography to afford the desired conjugates **5a**–**e** in moderate to high yields. The enantiomeric purity (>99% *ee*) of conjugates (*S*)**-5a** and (*S*)-**5e** was determined by reversed-phase chiral HPLC on a (*S*,*S*)-Whelk-O1 column (see the Supplementary Materials).

It is known that the presence of electron-donating or electron-withdrawing substituents determines the reactivity of pyrimidines [32]. 4,6-Dichloropyrimidines **4b** and **4c** are highly reactive due to the influence of the electron-withdrawing substituent, chlorine, at the *C*^6^ position. Their derivatives (*RS*)-**5b** and (*RS*)-**5c** were obtained in high yields of 68% (Scheme 1, entries 3 and 4). At the same time, the presence of electron-donating substituents in structures **4a** and **4e**, the amino group and the conjugated π-excess pyrrole ring, respectively, reduces the reactivity of these compounds; the yields of the products of nucleophilic substitution of chlorine (compounds **5a** and **5e**) were moderate (29–50%; Scheme 1, entries 1, 2, 6, and 7).

During the nucleophilic substitution of chlorine in dichloropyrimidines **4b** and **4c**, we did not observe the formation of by-products of double substitution, despite the excess of nucleophile **2**. This is due to the fact that the substitution of one of the chlorine atoms with an electron-donating alkylamino group deactivates another chlorine atom, and its substitution requires more drastic conditions [32]. The second chlorine atom can also be substituted in the presence of electron-withdrawing substituents in the pyrimidine ring [33,34].

### 2.2. Antiviral Evaluation

The antiviral activity of all synthesized pyrimidine conjugates with 7,8-difluoro-3,4-dihydro-3-methyl-2*H*-[1,4]benzoxazine attached via a linker (compounds **5a**–**e**) was tested in vitro against herpes simplex virus type 1 (HSV-1), including an acyclovir-resistant strain (Table 1), and influenza A (H1N1) virus (Table 2).

#### 2.2.1. Anti-Herpesvirus Activity

The anti-herpesvirus activity of the synthesized compounds **5a**–**e** was studied against the reference strain HSV-1/L_2_ and the acyclovir-resistant strain HSV-1/L_2_/R in the Vero E6 cells (Table 1) by suppressing the virus-induced cytopathogenic effect (CPE) as described previously [27]. Acyclovir, Foscarnet (sodium phosphonoformate hexahydrate), a drug of choice in clinical practice in case of inefficiency of acyclovir and related compounds, including cases of drug resistance, and Ribavirin, were used as reference drugs. To assess the cytotoxicity of the studied compounds, the percentage of viable and non-viable cells was determined using Trypan blue exclusion assay.

As can be seen from the data in Table 1, compounds **5a**–**e** exhibit low or very moderate antiviral activity against HSV-1. The most active is the 2-aminopyrimidine derivative (*S*)-**5a** containing the (*S*)-enantiomer of difluorobenzoxazine (IC_50_ = 9.27 µM); however, this compound possesses a high cytotoxicity against Vero E6 cells (CC_50_ = 34.63 µM), which results in a low antiviral selectivity (SI < 4). Compounds (*RS*)-**5a** and (*S*)-**5e** showed the highest selectivity of anti-herpesvirus activity (SI = 8 and 6, respectively); moreover, these indicators are preserved against the acyclovir-resistant HSV-1 strain. However, all the studied pyrimidine derivatives are inferior in antiviral activity and selectivity to the previously identified lead compound (*S*)-**1**. In general, it can be concluded that the replacement of a purine fragment with a pyrimidine one does not lead to an enhancement of anti-herpesvirus activity.

#### 2.2.2. Anti-Influenza Activity

The study of anti-influenza activity of the synthesized compounds **5a**–**e** was carried out against the reference strain of influenza virus A/Puerto Rico/8/34 (H1N1) using the test for the suppression of virus-induced CPE in the MDCK cell culture (Table 2), as described earlier [31]. The reference drug was Oseltamivir carboxylate, a drug used to treat influenza. The viability of infected and healthy cells was assessed using the MTT-assay [35].

The studied conjugates **5a**–**e** showed low activity against influenza A virus and low selectivity (Table 2). The conjugate of 2-amino-6-chloropyrimidine and racemic difluorobenzoxazine (compound (*RS*)-**5b**: CC_50_ = 705 µM, IC_50_ = 92 µM, SI < 8) had the optimal parameters of activity and cytotoxicity in a series of studied compounds. Compound (*RS*)-**5b**, although it showed a lower anti-influenza activity compared to the previously obtained compound (*S*)-**1** (IC_50_ > 10 µM), was less toxic to MDCK cells. 

## 3. Materials and Methods

### 3.1. Chemistry General Section

Racemic 7,8-difluoro-3,4-dihydro-3-methyl-4-(6-phthalimidohexanoyl)-2*H*-[1,4]benzoxazine [(*RS*)-**2**] and (*S*)-enantiomer [(*S*)-**2**] were obtained as previously described [23]. 2-Chloropyrimidine (**4d**) was obtained according to the known method [36] starting from 2-aminopyrimidine. Other reagents are commercially available and were purchased from Alfa Aesar (Lancashire, UK). Solvents were purified according to traditional methods [37] and used freshly distilled. Melting points were obtained on a SMP3 apparatus (Barloworld Scientific, Staffordshire, UK) and are uncorrected. Optical rotations were measured on a Perkin Elmer M341 polarimeter. The reactions were monitored by thin layer chromatography (TLC) using silica gel precoated Sorbfil plates (Imid, Krasnodar, Russia); compounds were visualized by UV irradiation at 254 nm and iodine vapors. Flash column chromatography was performed using silica gel 60 (230–450 mesh) (Alfa Aesar, UK). The ^1^H, ^19^F, and ^13^C NMR spectra were recorded on a Bruker AVANCE 500 spectrometer. Chemical shifts are given in ppm and are referenced to TMS and hexafluorobenzene as internal standards, and multiplicities are reported as s (singlet), d (doublet), t (triplet), q (quartet), and m (multiplet). The ^1^H and ^19^F NMR spectra of compounds **5a**–**c**,**e** were recorded in DMSO-*d*_6_ at 100 °C; other NMR spectra, at ambient temperature. CHN-Elemental analysis was performed using Perkin Elmer 2400 II analyser. High resolution mass spectra were obtained on a Bruker maXis Impact HD mass spectrometer (Bruker, Karlsruhe, Germany), electrospray ionization with direct sample inlet (4 L/min flow rate). Analytical chiral HPLC of compounds (*RS*)-**5a**, (*S*)-**5a**, (*RS*)-**5e**, and (*S*)-**5e** was performed on an Agilent 1100 instrument (Agilent Technologies, Santa Clara, CA, USA) using an (*S*,*S*)-Whelk-O1 column (250 × 4.6 mm, 5 µm) (Phenomenex, Torrance, CA, USA); flow rate 0.8 mL/min, detection at 280 nm.

### 3.2. General Procedure for the Synthesis of the Target Compounds ***5a**–**e***

Hydrazine monohydrate (0.22 mL, 4.50 mmol) was added to a solution of compound (*RS*)-**2** or (*S*)-**2** (1.07 g, 2.50 mmol) in ethanol (25 mL). The reaction mixture was refluxed for 2 h and then evaporated to dryness under reduced pressure. The residue was stirred in 2 M HCl (25 mL) at +4 °C for 12 h. The precipitate was filtered off, washed with 1 M HCl (2 × 3 mL). The filtrate and washings were combined and neutralized with solid NaOH to pH 9, then extracted with chloroform (3 × 5 mL). The organic layers were dried with MgSO_4_ and evaporated to dryness under reduced pressure to afford compound (*RS*)-**3** or (*S*)-**3** (0.56 g, 75%). The residue was dissolved in *n*BuOH (8 mL). The appropriate chloropyrimidine **4a**–**e** (1.25 mmol) and Et_3_N (0.17 mL, 1.25 mmol) were added to the resulting solution. The reaction mixture was refluxed for 8 h, cooled to room temperature, diluted with *n*BuOH (12 mL), washed with 1 M HCl (in the case of pyrimidines **4a,b**, with 10% aqueous citric acid) (3 × 5 mL), saturated aqueous NaCl solution (3 × 5 mL), and water (3 × 3 mL), then evaporated to dryness under reduced pressure. The residue was purified by flash column chromatography on silica gel using the appropriate eluent.

*(RS)-4-[6-(2-Aminopyrimidin-4-yl)aminohexanoyl]-7,8-difluoro-3,4-dihydro-3-methyl-2H-*[1,4]*benzoxazine Sesquihydrate* [(*RS*)-**5a**]. White powder m.p. 59–60 °C (yield 0.22 g, 44%) after flash column chromatography, chloroform–EtOH 85: 15 as an eluent. HPLC ((*S*,*S*)-Whelk-O1, MeOH–0.2% aqueous Et_3_N 9: 1, 0.8 mL/min; detection at 280 nm): *τ*_(*S*)_ 8.0 min, *τ*_(*R*)_ 9.8 min. ^1^H NMR (500 MHz, DMSO-*d*_6_, 100 °C) *δ* (ppm): 1.13 (d, *J* = 6.9 Hz, 3H, Me), 1.34–1.40 (m, 2H, CH_2_ hexanoyl), 1.52–1.57 (m, 2H, CH_2_ hexanoyl), 1.59–1.66 (m, 2H, CH_2_ hexanoyl), 2.44–2.50 (m, 1H, 2-H_B_ hexanoyl, partly overlapped with DMSO signal), 2.58–2.64 (m, 1H, 2-H_A_ hexanoyl), 3.28 (td, *J* = 6.7, 6.2 Hz, 2H, 6-H hexanoyl), 4.14 (dd, *J* = 10.9, 2.8 Hz, 1H, 2-H_B_ benzoxazine), 4.33 (d, *J* = 11.1 Hz, 1H, 2-H_A_ benzoxazine), 4.74 (q, *J* = 6.1 Hz, 1H, 3-H benzoxazine), 5.94 (d, *J* = 6.7 Hz, 1H, 5-H pyrimidine), 6.59 (br. s, 2H, NH_2_), 6.85 (dd, *J* = 9.7, 8.5 Hz, 1H, 6-H benzoxazine), 7.54–7.57 (m, 1H, 5-H benzoxazine), 7.59 (br. s, 1H, NH), 7.60 (br. s, 1H, 6-H pyrimidine). ^19^F NMR (470 MHz, DMSO-*d*_6_, 100 °C) *δ* (ppm): 2.05 (dd, *J* = 20.8, 6.7 Hz, 1F, 8-F), 20.18 (br. s, 1F, 7-F). ^13^C NMR (126 MHz, DMSO-*d*_6_) *δ* (ppm): 15.10, 24.33, 25.95, 28.35, 33.37, 39.6 (overlapped by DMSO signal), 44.75 (br. s), 69.85, 96.95 (br. s), 106.77 (d, *J* = 17.9 Hz), 119.25, 121.78, 135.69 (br. d, *J* = 9.7 Hz), 138.91 (dd, *J* = 243.3, 15.3 Hz), 145.52 (br. s), 146.49 (br. d, *J* = 241.6 Hz), 158.50, 162.67, 170.91. Calcd (%) for C_19_H_23_F_2_N_5_O_2_ × 1.5H_2_O: C 54.54, H 6.26, N 16.74, F 9.08. Found (%): C 54.33, H 6.13, N 16.37, F 9.03.

*(S)-4-[6-(2-Aminopyrimidin-4-yl)aminohexanoyl]-7,8-difluoro-3,4-dihydro-3-methyl-2H-*[1,4]*benzoxazine Hydrate* [(*S*)-**5a**]. White powder m.p. 78–80 °C (yield 0.18 g, 36%) after flash column chromatography, chloroform–EtOH 85: 15 as an eluent. [α]_D_^20^ +60.5 (*c* 0.5, EtOH). >99% *ee*. HPLC ((*S*,*S*)-Whelk-O1, MeOH–0.2% aqueous Et_3_N 9: 1, 0.8 mL/min; detection at 280 nm): *τ*_(*S*)_ 8.0 min. ^1^H, ^19^F, and ^13^C NMR spectra were identical to those of compound (*RS*)-**5a**. Calcd (%) for C_19_H_23_F_2_N_5_O_2_ × 1.25H_2_O: C 55.13, H 6.21, N 16.92. Found (%): C 55.07, H 6.29, N 16.62.

*(RS)-4-[6-(2-Amino-6-chloropyrimidin-4-yl)aminohexanoyl]-7,8-difluoro-3,4-dihydro-3-methyl-2H-*[1,4]*benzoxazine* [(*RS*)-**5b**]. Yellowish powder m.p. 92–94 °C (yield 0.36 g, 68%) after flash column chromatography, chloroform–EtOH 97: 3 as an eluent. HPLC ((*S,S*)-Whelk-O1, MeOH–H_2_O 75: 25, 0.8 mL/min; detection at 280 nm): *τ*_1_ 23.7 min, *τ*_2_ 31.5 min. ^1^H NMR (500 MHz, DMSO-*d*_6_, 100 °C) *δ* (ppm): 1.12 (d, *J* = 6.9 Hz, 3H, Me), 1.33–1.38 (m, 2H, CH_2_ hexanoyl), 1.48–1.54 (m, 2H, CH_2_ hexanoyl), 1.57–1.66 (m, 2H, CH_2_ hexanoyl), 2.44–2.52 (m, 1H, 2-H_B_ hexanoyl, partly overlapped with DMSO signal), 2.57–2.64 (m, 1H, 2-H_A_ hexanoyl), 3.20 (td, *J* = 6.7, 5.9 Hz, 2H, 6-H hexanoyl), 4.14 (dd, *J* = 10.9, 2.8 Hz, 1H, 2-H_B_ benzoxazine), 4.33 (dd, *J* = 11.0, 1.5 Hz, 1H, 2-H_A_ benzoxazine), 4.73 (q, *J* = 6.9 Hz, 1H, 3-H benzoxazine), 5.74 (s, 1H, 5-H pyrimidine), 5.88 (br. s, 2H, NH_2_), 6.69 (br. s, 1H, NH), 6.85 (ddd, *J* = 9.8, 8.3, 5.7 Hz, 1H, 6-H benzoxazine), 7.56 (ddd, *J* = 11.0, 6.8, 2.8 Hz, 1H, 5-H benzoxazine). ^19^F NMR (470 MHz, DMSO-*d*_6_, 100 °C) *δ* (ppm): 2.01 (ddd, *J* = 21.0, 8.2, 2.1 Hz, 1F, 8-F), 20.14 (br. s, 1F, 7-F). ^13^C NMR (126 MHz, DMSO-*d*_6_) *δ* (ppm): 15.10, 24.38, 25.98, 28.67, 33.40, 39.6 (overlapped by DMSO signal), 45.12 (br. s), 69.84, 92.58 (br. s), 106.77 (d, *J* = 17.9 Hz), 119.26, 121.78, 135.68 (br. d, *J* = 9.7 Hz), 138.92 (dd, *J* = 243.3, 15.3 Hz), 146.47 (br. d, *J* = 241.6 Hz), 157.05 (br. s), 162.88, 164.00, 170.93. Calcd (%) for C_19_H_22_ClF_2_N_5_O_2_: C 53.59, H 5.21, N 16.45, Cl 8.33, F 8.92. Found (%): C 53.53, H 5.33, N 16.32, Cl 8.10, F 9.27.

*(RS)-4-[6-(6-Chloropyrimidin-4-yl)aminohexanoyl]-7,8-difluoro-3,4-dihydro-3-methyl-2H-*[1,4]*benzoxazine* [(*RS*)-**5c**]. White powder m.p. 101–103 °C (yield 0.35 g, 68%) after flash column chromatography, EtOAc as an eluent. HPLC ((*S*,*S*)-Whelk-O1, MeOH–H_2_O 75: 25, 0.8 mL/min; detection at 254 nm): *τ*_1_ 21.6 min, *τ*_2_ 28.7 min. ^1^H NMR (500 MHz, DMSO-*d*_6_, 100 °C) *δ* (ppm): 1.12 (d, *J* = 6.9 Hz, 3H, Me), 1.34–1.40 (m, 2H, CH_2_ hexanoyl), 1.51–1.57 (m, 2H, CH_2_ hexanoyl), 1.59–1.64 (m, 2H, CH_2_ hexanoyl), 2.44–2.50 (m, 1H, 2-H_B_ hexanoyl, overlapped with DMSO signal), 2.57–2.63 (m, 1H, 2-H_A_ hexanoyl), 3.27 (td, *J* = 6.7, 6.2 Hz, 2H, 6-H hexanoyl), 4.13 (dd, *J* = 10.9, 2.9 Hz, 1H, 2-H_B_ benzoxazine), 4.33 (dd, *J* = 10.9, 1.4 Hz, 1H, 2-H_A_ benzoxazine), 4.74 (qd, *J* = 6.9, 2.9 Hz, 1H, 3-H benzoxazine), 6.47 (s, 1H, 5-H pyrimidine), 6.84 (ddd, *J* = 9.9, 9.6, 8.3 Hz, 1H, 6-H benzoxazine), 7.31 (br. s, 1H, NH), 7.55 (ddd, *J* = 9.3, 5.4, 2.4 Hz, 1H, 5-H benzoxazine), 8.19 (s, 1H, 2-H pyrimidine). ^19^F NMR (470 MHz, DMSO-*d*_6_, 100 °C) *δ* (ppm): 2.02 (ddd, *J* = 21.0, 8.1, 2.3 Hz, 1F, 8-F), 20.09–20.17 (m, 1F, 7-F). ^13^C NMR (126 MHz, DMSO-*d*_6_) *δ* (ppm): 15.17, 24.40, 25.96, 28.53, 33.42, 39.6 (overlapped by DMSO signal), 44.88 (br. s), 69.91, 103.53, 106.83 (d, *J* = 17.9 Hz), 119.33, 121.85, 135.75 (br. d, *J* = 9.7 Hz), 138.99 (dd, *J* = 243.3, 15.4 Hz), 146.55 (br. d, *J* = 243.9 Hz), 156.86 (br. s), 158.56, 163.19, 170.97. Calcd (%) for C_19_H_21_ClF_2_N_4_O_2_: C 55.54, H 5.15, N 13.64, Cl 8.63, F 9.25. Found (%): C 55.44, H 5.01, N 13.58, Cl 8.61, F 8.89.

*(RS)-7,8-Difluoro-3,4-dihydro-3-methyl-4-[6-(pyrimidin-2-yl)aminohexanoyl]-2H-*[1,4]*benzoxazine* [(*RS*)-**5d**]. White powder m.p. 106–108 °C (yield 0.41 g, 87%) after flash column chromatography, benzene–EtOAc–MeOH 7: 3: 0.2 as an eluent. ^1^H NMR (500 MHz, DMSO-*d*_6_) *δ* (ppm): 1.11 (d, *J* = 5.8 Hz, 3H, Me), 1.28–1.40 (m, 2H, CH_2_ hexanoyl), 1.49–1.64 (m, 4H, CH_2_–CH_2_ hexanoyl), 2.43-2.50 (m, 1H, 2-H_B_ hexanoyl, partly overlapped with DMSO signal), 2.62-2.68 (m, 1H, 2-H_A_ hexanoyl), 3.23 (td, *J* = 6.6, 6.5 Hz, 2H, 6-H hexanoyl), 4.14 (d, *J* = 10.3 Hz, 1H, 2-H_B_ benzoxazine), 4.35 (d, *J* = 10.7 Hz, 1H, 2-H_A_ benzoxazine), 4.71 (br. s, 1H, 3-H benzoxazine), 6.51 (dd, *J* = 4.7, 4.7 Hz, 1H, 5-H pyrimidine), 6.92 (dd, *J* = 9.5, 8.8 Hz, 1H, 6-H benzoxazine), 7.09 (t, *J* = 5.5 Hz, 1H, NH hexanoyl), 7.40-8.10 (m, 1H, 5-H benzoxazine), 8.23 (d, *J* = 4.6 Hz, 2H, 4-H and 6-H pyrimidine). ^19^F NMR (470 MHz, DMSO-*d*_6_,) *δ* (ppm): 1.85 (br. s, 1F, 8-F), 19.53 (br. s, 1F, 7-F). ^13^C NMR (126 MHz, DMSO-*d*_6_,) *δ* (ppm): 15.07, 24.41, 25.97, 28.68, 33.39, 40.36, 44.73 (br. s), 69.83, 106.73 (d, *J* = 17.9 Hz), 109.54, 119.24, 121.78, 135.66 (dd, *J* = 9.8, 2.4 Hz), 138.90 (dd, *J* = 243.2, 15.4 Hz), 146.45 (dd, *J* = 242.0, 10.5 Hz), 157.73 (2C), 162.27, 170.93. Calcd (%) for C_19_H_22_F_2_N_4_O_2_: C 60.63, H 5.89, N 14.88. Found (%): C 60.60, H 6.02, N 14.71.

*(RS)-7,8-Difluoro-3,4-dihydro-3-methyl-4-[6-(7H-pyrrolo[2,3-d]pyrimidin-4-yl)aminohexanoyl]-2H-*[1,4]*benzoxazine Hemihydrate* [(*RS*)-**5e**]. White powder m.p. 86–88 °C (yield 0.26 g, 50%) after flash column chromatography, EtOAc–EtOH 9: 1 as an eluent. HPLC ((*S*,*S*)-Whelk-O1, MeOH–H_2_O 75: 25, 0.8 mL/min; detection at 280 nm): *τ*_(*S*)_ 22.4 min, *τ*_(*R*)_ 29.0 min. ^1^H NMR (500 MHz, DMSO-*d*_6_, 100 °C) *δ* (ppm): 1.12 (d, *J* = 6.8 Hz, 3H, Me), 1.39–1.44 (m, 2H, CH_2_ hexanoyl), 1.60–1.68 (m, 4H, CH_2_–CH_2_ hexanoyl), 2.45–2.51 (m, 1H, 2-H_B_ hexanoyl, partly overlapped with DMSO signal), 2.58–2.64 (m, 1H, 2-H_A_ hexanoyl), 3.47 (td, *J* = 6.9, 6.1 Hz, 2H, 6-H hexanoyl), 4.13 (dd, *J* = 10.9, 2.8 Hz, 1H, 2-H_B_ benzoxazine), 4.32 (dd, *J* = 10.9, 1.3 Hz, 1H, 2-H_A;_ benzoxazine), 4.73 (q, *J* = 5.6 Hz, 1H, 3-H benzoxazine), 6.50 (d, *J* = 3.3 Hz, 1H, 5-H pyrrole), 6.83 (dd, *J* = 9.8, 8.4 Hz, 1H, 6-H benzoxazine), 6.87 (br. s, 1H, NH hexanoyl), 6.97 (d, *J* = 3.4 Hz, 1H, 4-H pyrrole), 7.54-7.57 (m, 1H, 5-H benzoxazine), 8.05 (s, 1H, 2-H pyrimidine), 11.05 (s, 1H, NH pyrrole). ^19^F NMR (470 MHz, DMSO-*d*_6_, 100 °C) *δ* (ppm): 1.99 (ddd, *J* = 21.1, 8.3, 2.1 Hz, 1F, 8-F), 20.09 (br. s, 1F, 7-F). ^13^C NMR (126 MHz, DMSO-*d*_6_) *δ* (ppm): 15.09, 24.47, 26.08, 29.09, 33.43, 39.6 (overlapped by DMSO signal), 44.87 (br. s), 69.84, 98.48, 102.37, 106.75 (d, *J* = 17.9 Hz), 119.26, 120.45, 121.79, 135.68 (dd, *J* = 9.7, 1.9 Hz), 138.92 (dd, *J* = 243.2, 15.3 Hz), 146.50 (br. d, *J* = 241.8 Hz), 149.99, 151.41, 156.09, 170.95. Calcd (%) for C_21_H_23_F_2_N_5_O_2_ × 0.5H_2_O: C 59.43, H 5.70, N 16.50, F 8.95. Found (%): C 59.59, H 5.76, N 16.40, F 8.58.

*(3S)-7,8-Difluoro-3,4-dihydro-3-methyl-4-[6-(7H-pyrrolo[2,3-d]pyrimidin-4-yl)aminohexanoyl]-2H-*[1,4]*benzoxazine* [(*S*)-**5e**]. White powder m.p. 87–89 °C (yield 0.15 g, 29%) after flash column chromatography, EtOAc–EtOH 9: 1 as an eluent. [α]_D_^20^ +57.5 (*c* 0.5, EtOH). >99% *ee*. HPLC ((*S*,*S*)-Whelk-O1, MeOH–H_2_O 75: 25, 0.8 mL/min; detection at 280 nm): *τ*_(*S*)_ 22.3 min. ^1^H, ^19^F and ^13^C NMR spectra were identical to those of compound (*RS*)-**5e**. HRMS (ESI): *m/z* [M+H]^+^ calcd for [C_21_H_2__4_F_2_N_5_O_2_]^+^: 416.1898, found: 416.1897.

## 4. Conclusions

In summary, we have synthesized a series of pyrimidine conjugates containing a fragment of both racemic 7,8-difluoro-3,4-dihydro-3-methyl-2*H*-[1,4]benzoxazine and its (*S*)-enantiomer, which are attached via a 6-aminohexanoyl fragment. The structure of the synthesized compounds was confirmed by ^1^H, ^19^F, and ^13^C NMR spectroscopy data. Chiral HPLC was used to confirm the enantiomeric purity of the optically active derivatives. Evaluation of the antiviral activity of the synthesized compounds has shown that the replacement of purine with a fragment of pyrimidines and aminopyrimidines leads to a decrease in the anti-herpesvirus activity compared to the lead compound. The studied compounds did not exhibit significant activity against influenza A (H1N1) virus.

## Figures and Tables

**Table 1:** Cytotoxicity and inhibitory activity of compounds **5a**–**e** against herpes simplex virus type 1 (HSV-1) strains in the Vero E6 cells.

Compound	CC_50_ ± SD (µM)	HSV-1/L_2_		HSV-1/L_2_/R	
		IC_50_ (µM)	SI	IC_50_ (µM)	SI
(*S*)-**1**	293.48 ± 15.55	4.6	64	4.6	64 ^1^
(*RS*)-**5a**	150.56 ± 8.36	18.56	8.1	18.56	8
(*S*)-**5a**	34.63 ± 0.88	9.27	3.7	9.27	<4
(*RS*)-**5b**	274.57 ± 4.30	73.38	3.7	73.38	<4
(*RS*)-**5c**	>304.25	148.44	>2.0	152.12	2
(*RS*)-**5d**	504.77 ± 17.72	296.88	1.7	296.88	<2
(*RS*)-**5e**	17.58 ± 0.85	18.56	<1	18.56	<1
(*S*)-**5e**	468.97 ± 10.83	74.22	6.3	75.22	6
Acyclovir	>444	1.73	>256	>444	1
Foscarnet	>416.61	104.15	>4	104.15	>4
Ribavirin	>4098	1025	>4	1025	>4

^1^ Data from [27] for comparison. Multiplicity of infection 0.1 PFU/cell; CC_50_ values are averages with standard deviations for three independent experiments; IC_50_ concentration of compounds producing 50% inhibition of the development of the virus-induced CPE relative to complete CPE in infected but untreated control cultures; SI, selectivity index calculated as CC_50_/IC_50_.

**Table 2:** Cytotoxicity and antiviral activity of compounds **5a**–**e** against influenza A/Puerto Rico/8/34 (H1N1) virus in the MDCK cell culture.

Compound	CC_50_ ± SD (µM)	IC_50_ (µM)	SI
(*S*)-**1**	24 ± 1	>10	>2 ^1^
(*RS*)-**5a**	11 ± 1	8 ± 1	>1
(*S*)-**5a**	23 ± 1.2	7 ± 1	>3
(*RS*)-**5b**	705 ± 42	92 ± 11	<8
(*RS*)-**5c**	9 ± 1	>8	1
(*RS*)-**5d**	257 ± 12	>88	3
(*RS*)-**5e**	86 ± 4	>80	1
(*S*)-**5e**	47 ± 3	>26	<2
Oseltamivir carboxylate	>200	0.3	>667

^1^ Data from [31] for comparison. Multiplicity of infection 0.01 TCID_50_/cell; CC_50_ values are averages with standard deviations for three independent experiments; IC_50_ concentration of compounds producing 50% inhibition of the development of the virus-induced CPE relative to complete CPE in infected but untreated control cultures; SI, selectivity index calculated as CC_50_/IC_50_.

## Data Availability

Not applicable.

## Supplementary Materials

The following supporting information can be downloaded at: www.mdpi.com/article/10.3390/molecules27134236/s1, NMR spectra and HPLC data.
