# Supplementary material for: Synthesis of Pyrimidine Conjugates with 4-(6-Amino-hexanoyl)-7,8-difluoro-3,4-dihydro-3-methyl-2H-[1,4]benzoxazine and Evaluation of Their Antiviral Activity
Source: Molecules. 2022 Jun 30;27(13):4236. doi: 10.3390/molecules27134236 (PMC9268552; doi:10.3390/molecules27134236)
Supplement: Supplementary file 1 [file molecules-27-04236-s001.zip › molecules-1794252-supplementary.pdf]

Supplementary Materials

Table of Contents

NMR Spectra S-1

HPLC Data... S-7

NMR Spectra

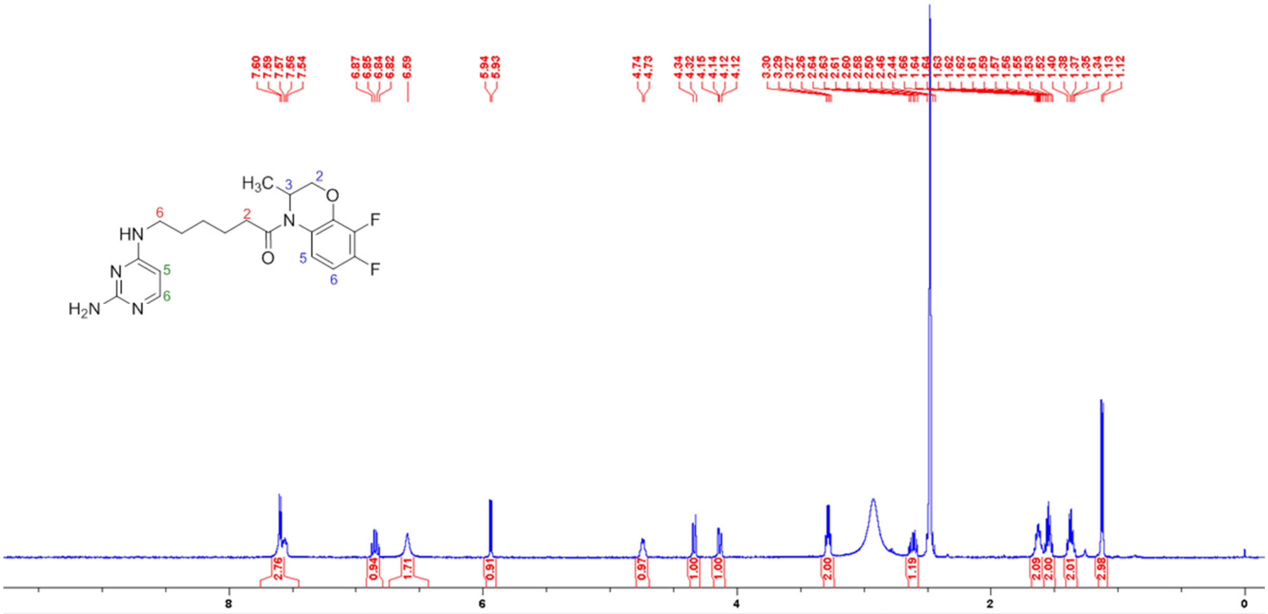

^{1}H NMR spectrum of compound (*RS*)-**5a** (500 MHz, DMSO-d_6, 100 °C)

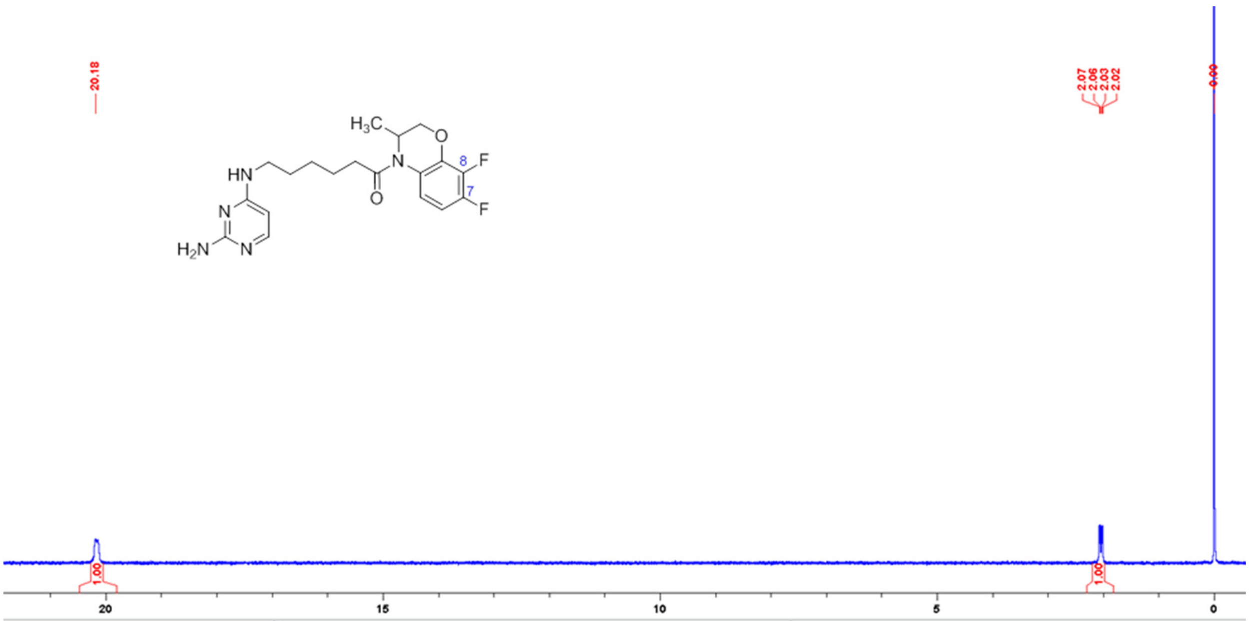

^{19}F NMR spectrum of compound (*RS*)-**5a** (470 MHz, DMSO-d_6, 100 °C)

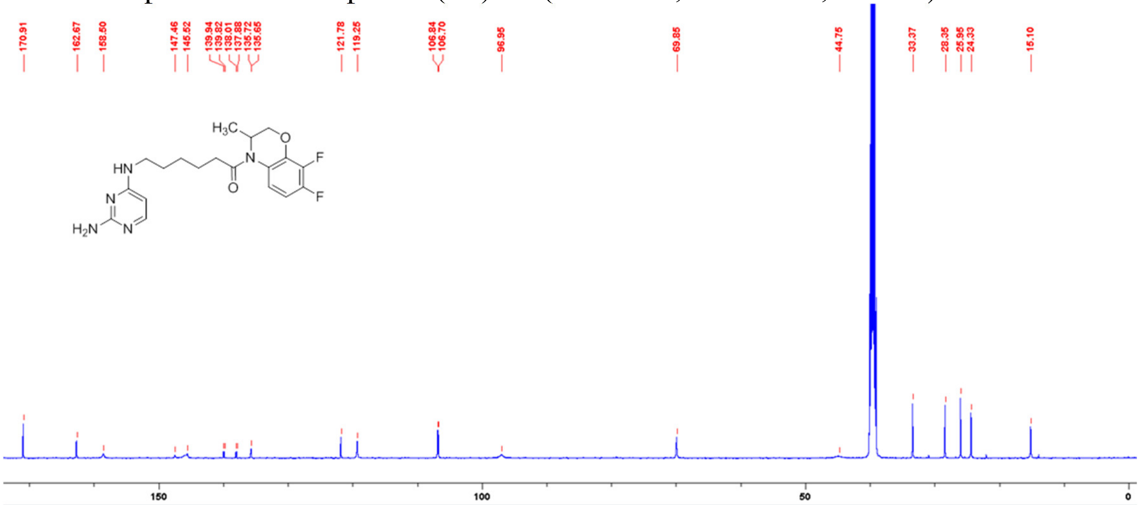

^{13}C NMR spectrum of compound (*RS*)-**5a** (126 MHz, DMSO-d_6)

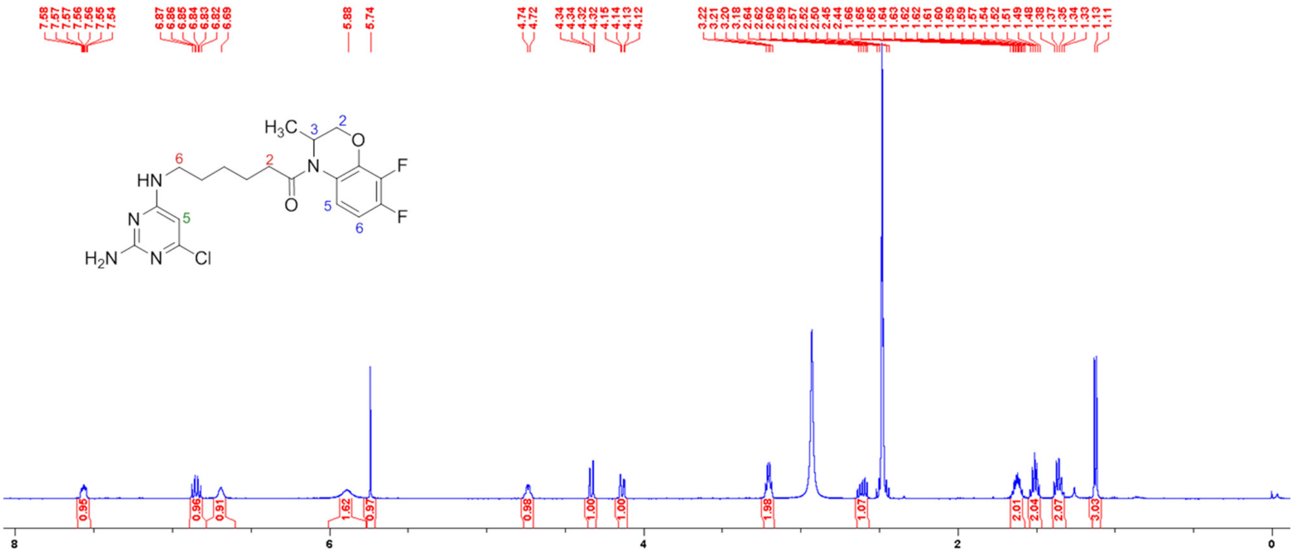

^{1}H NMR spectrum of compound (*RS*)-**5b** (500 MHz, DMSO-d_6, 100 °C)

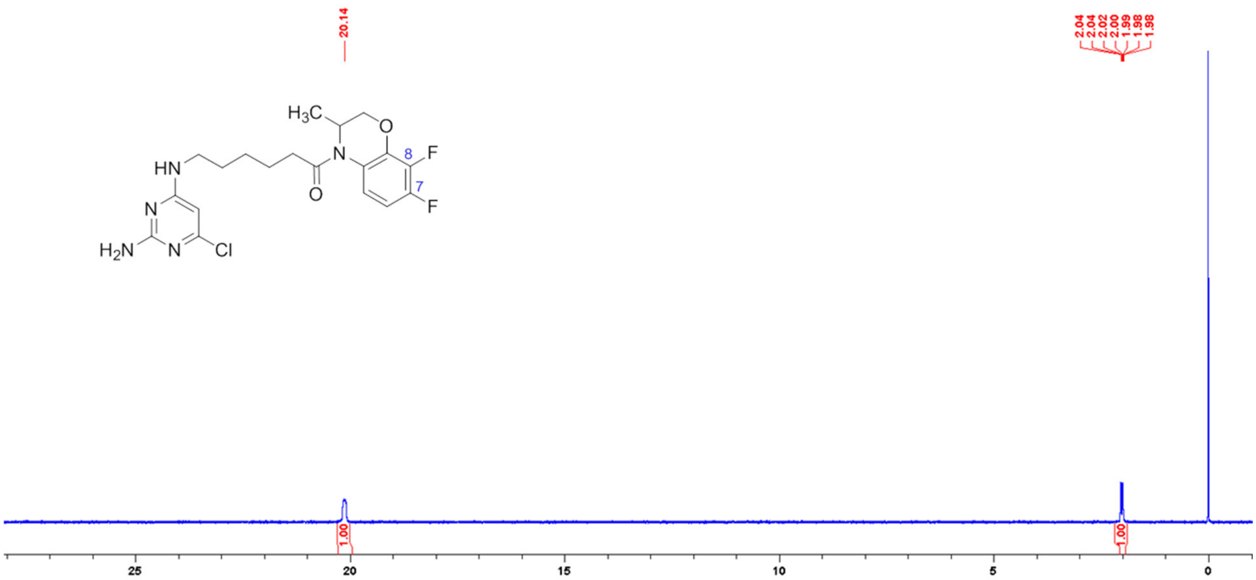

^{19}F NMR spectrum of compound (*RS*)-**5b** (470 MHz, DMSO-d_6, 100 °C)

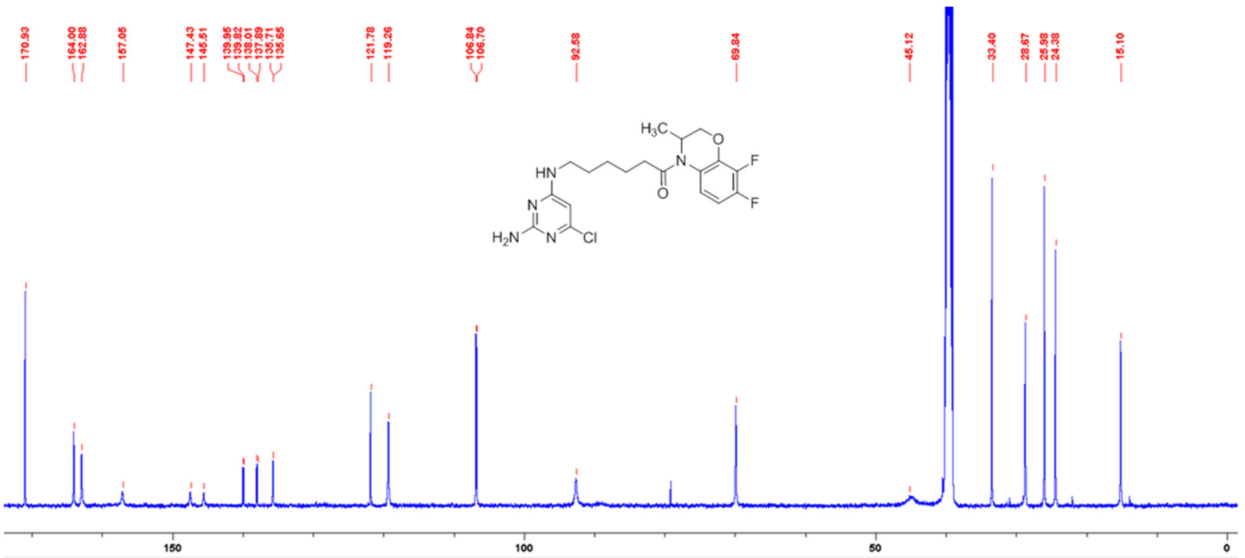

^{13}C NMR spectrum of compound (*RS*)-**5b** (126 MHz, DMSO-d_6)

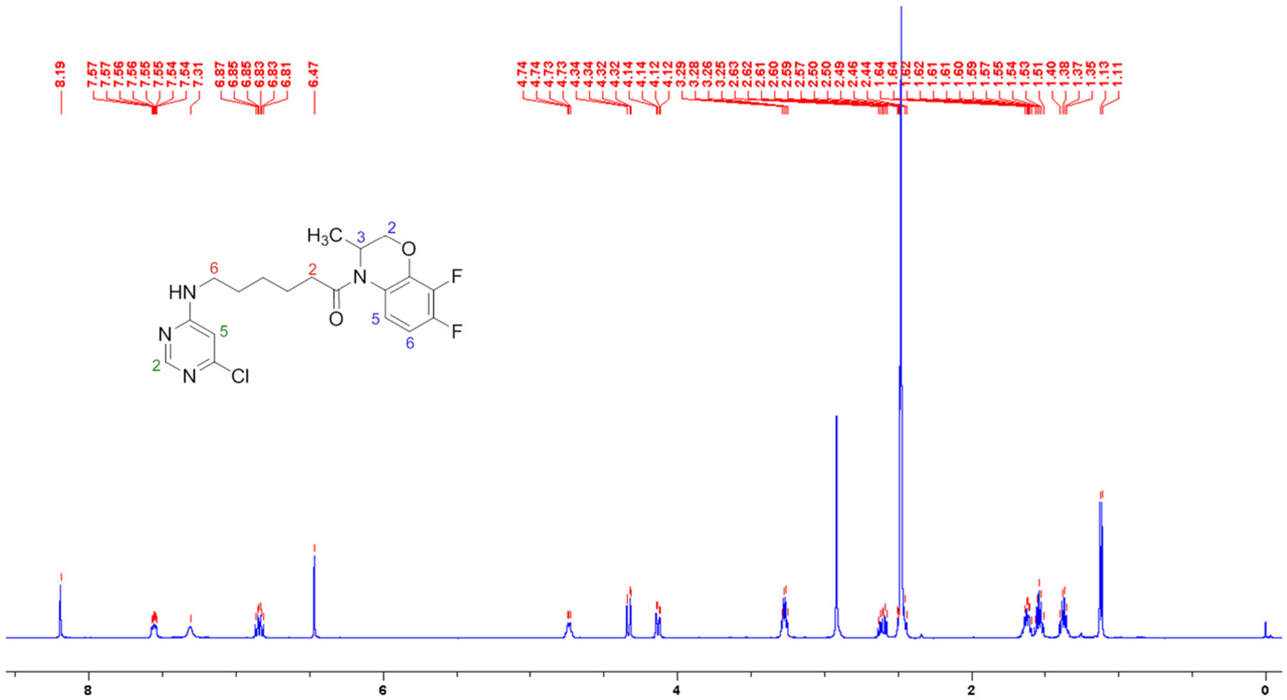

^{1}H NMR spectrum of compound (*RS*)-**5c** (500 MHz, DMSO-d_6, 100 °C)

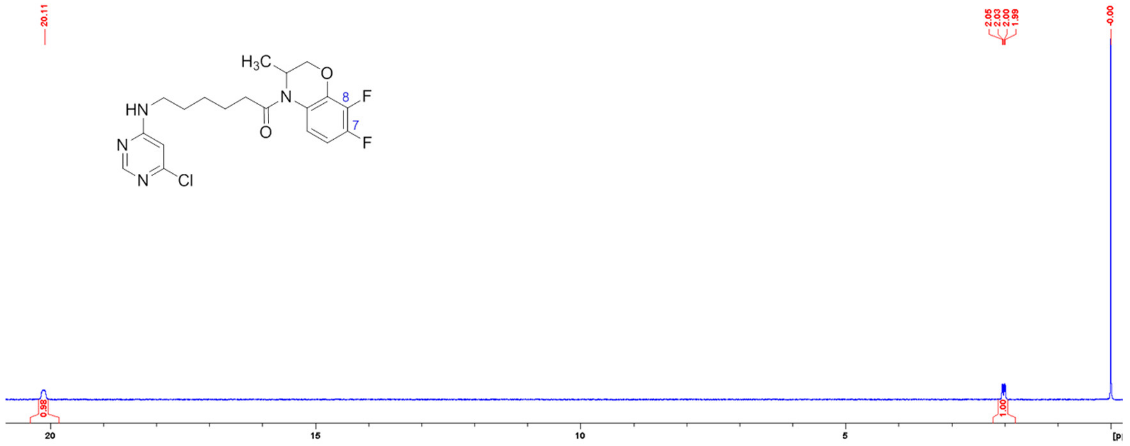

^{19}F NMR spectrum of compound (*RS*)-**5c** (470 MHz, DMSO-d_6, 100 °C)

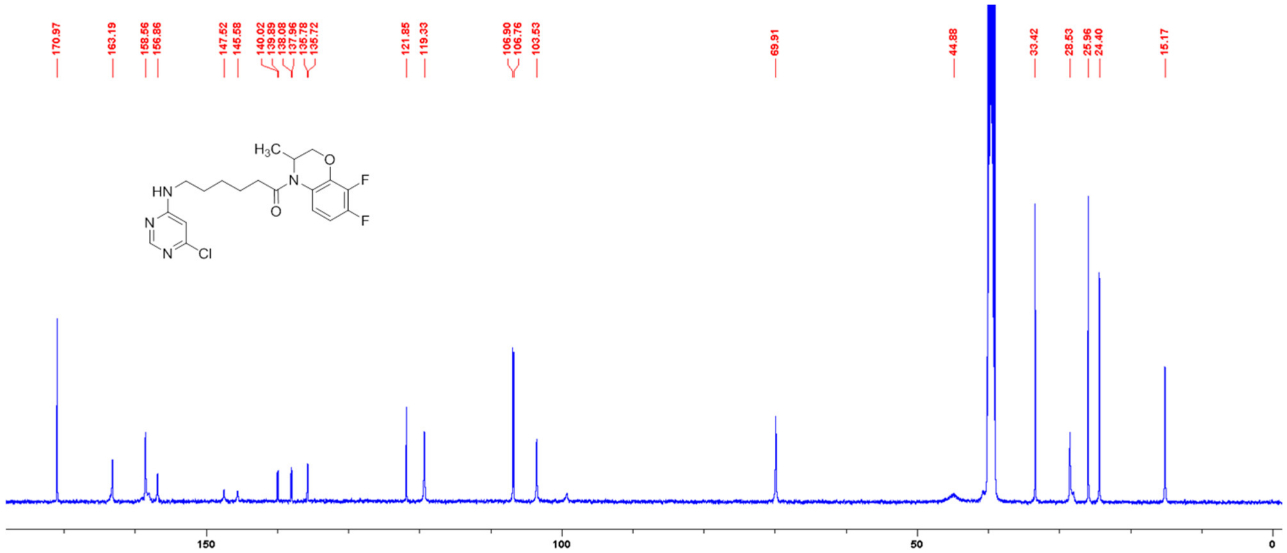

^{13}C NMR spectrum of compound (*RS*)-**5c** (126 MHz, DMSO-d_6)

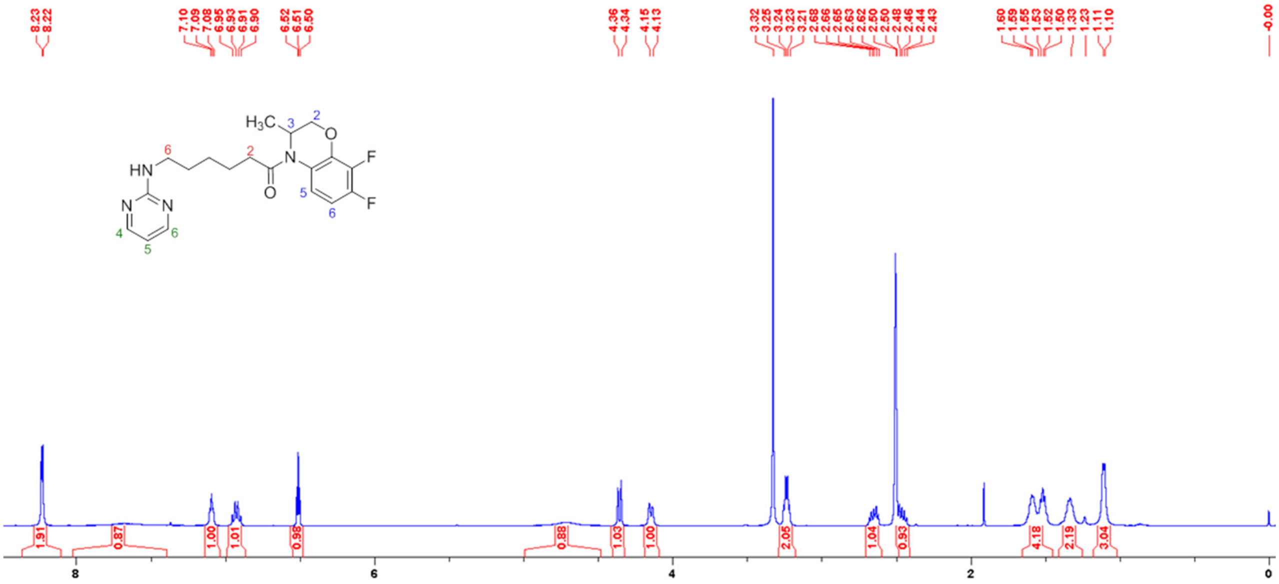

^{1}H NMR spectrum of compound (*RS*)-**5d** (500 MHz, DMSO-d_6)

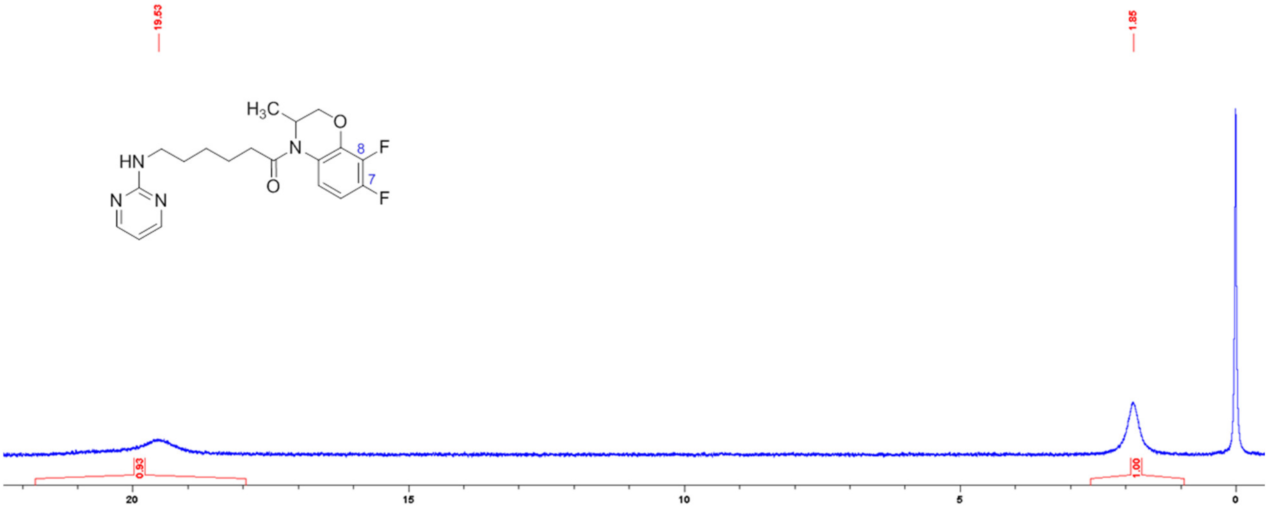

^{19}F NMR spectrum of compound (*RS*)-**5d** (470 MHz, DMSO-d_6)

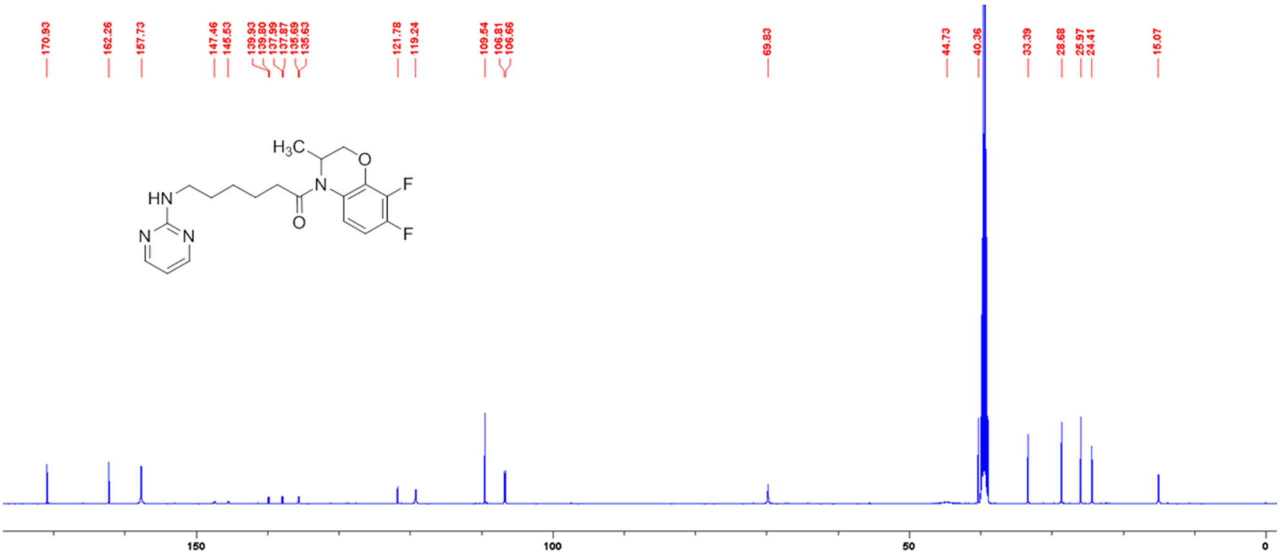

^{13}C NMR spectrum of compound (*RS*)-**5d** (126 MHz, DMSO-d_6)

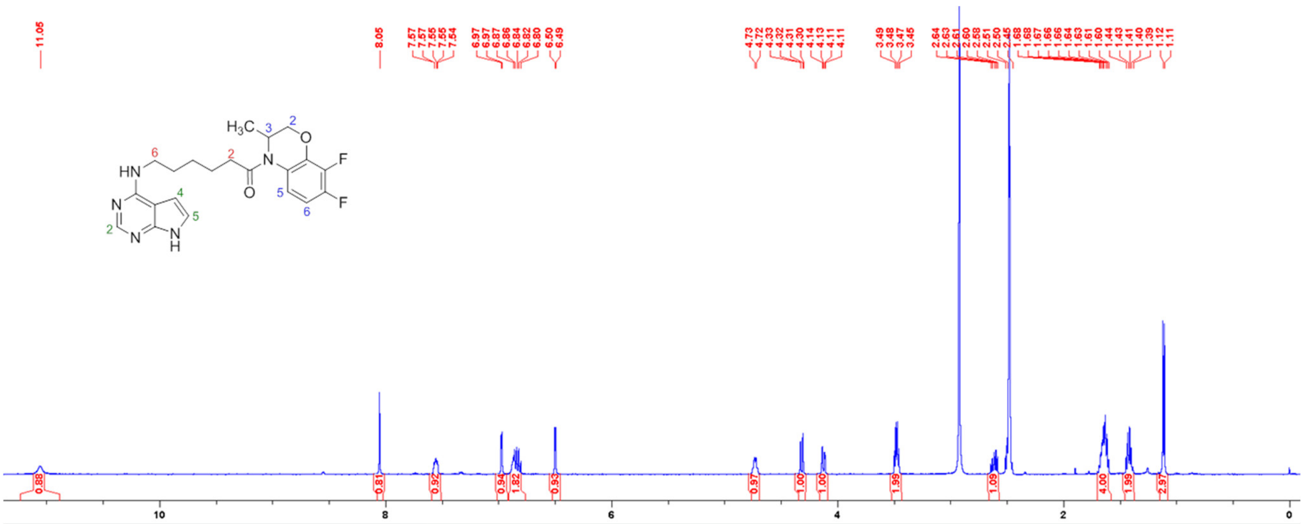

^{1}H NMR spectrum of compound (*RS*)-**5e** (500 MHz, DMSO-d_6, 100 °C)

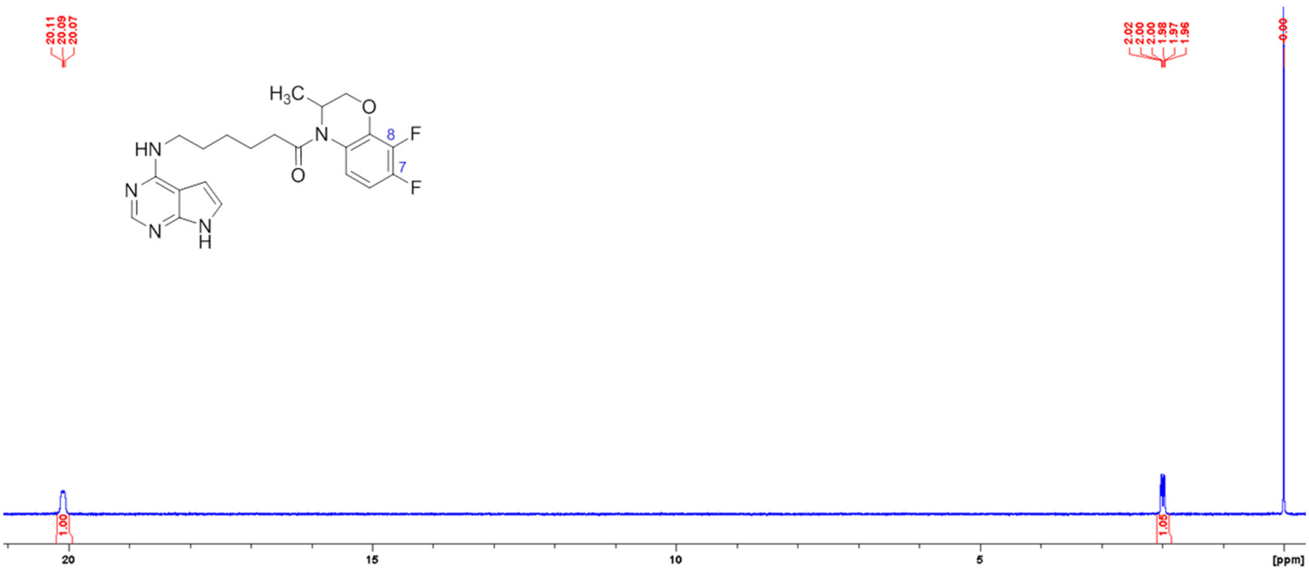

^{19}F NMR spectrum of compound (*RS*)-**5e** (470 MHz, DMSO-d_6, 100 °C)

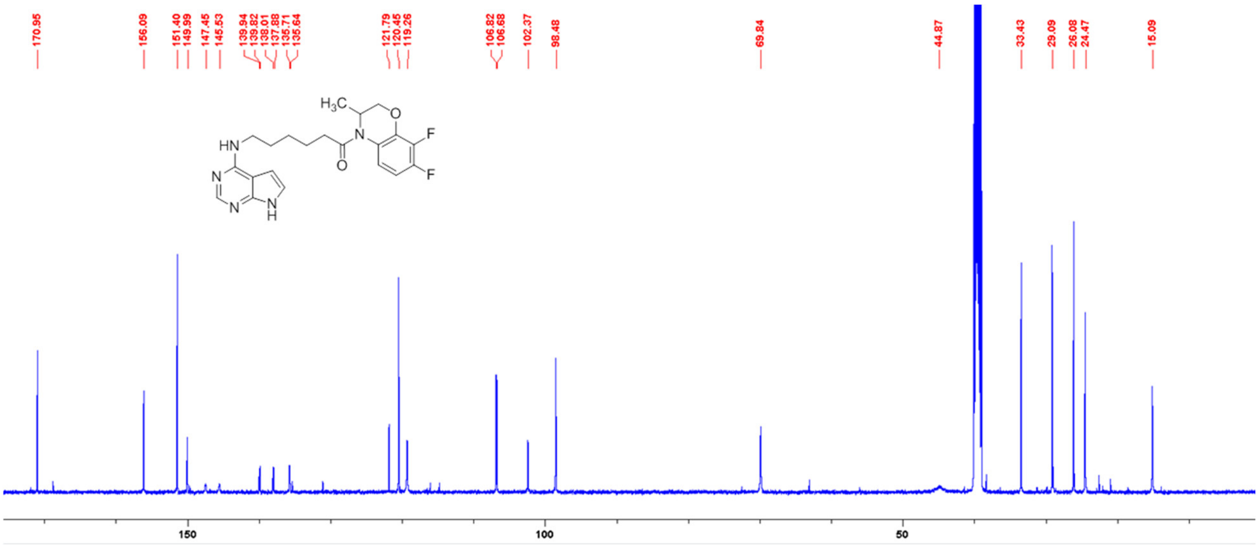

^{13}C NMR spectrum of compound (*RS*)-**5e** (126 MHz, DMSO-d_6)

HPLC data

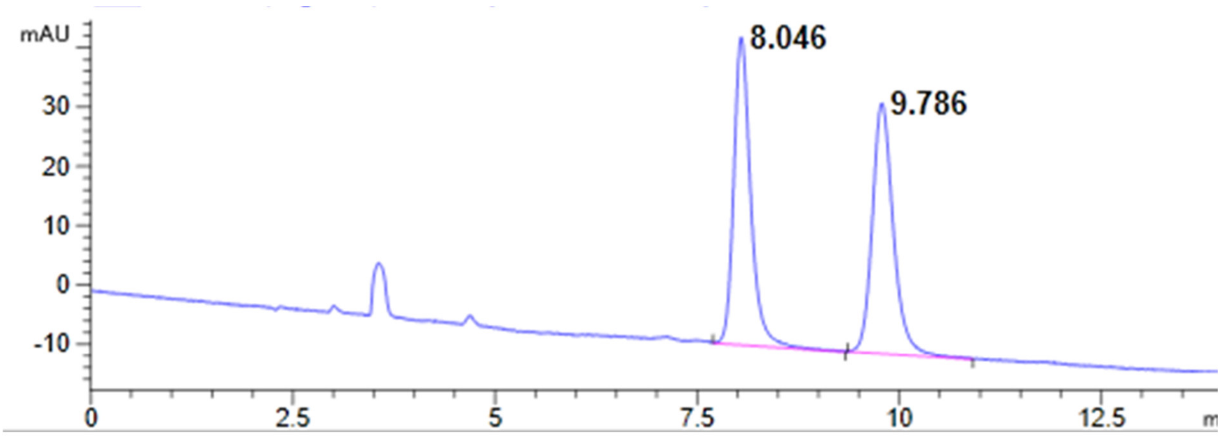

Peak #	RetTime [min]	Type	Width [min]	Area [mAU*s]	Height [mAU]	Area %
1	8.046	BB	0.2210	759.91211	51.84130	49.7168
2	9.786	BB	0.2725	768.56995	42.20139	50.2832

HPLC of compound (*RS*)-**5a** (*S*,*S*)-Whelk-O1, MeOH–0.2% aqueous Et_3N 9 : 1, 0.8 mL/min; detection at 280 nm): $\tau_{(S)}$ = 8.05 min, $\tau_{(R)}$ = 9.79 min

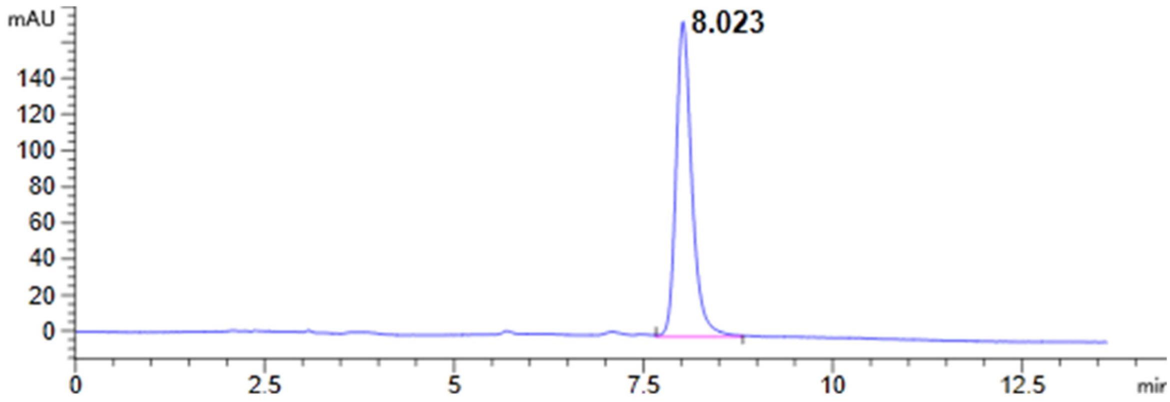

Peak #	RetTime [min]	Type	Width [min]	Area [mAU*s]	Height [mAU]	Area %
1	8.023	MM	0.2433	2549.60498	174.63181	100.0000

HPLC of compound (*S*)-**5a** (*S*,*S*)-Whelk-O1, MeOH–0.2% aqueous Et_3N 9 : 1, 0.8 mL/min; detection at 280 nm): $\tau_{(S)}$ = 8.02 min (> 99% *ee*)

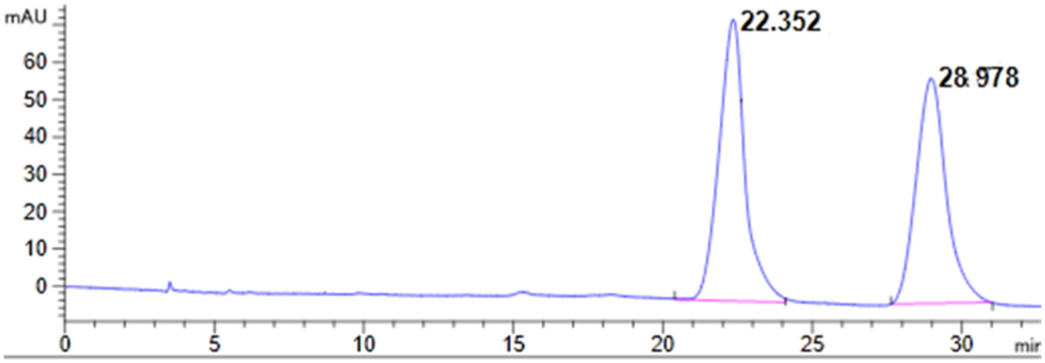

Peak #	RetTime [min]	Type	Width [min]	Area [mAU*s]	Height [mAU]	Area %
1	22.352	MM	1.0168	4610.15967	75.56788	52.0531
2	28.978	MM	1.1720	4246.48926	60.38737	47.9469

HPLC of compound (*RS*)-**5e** ((*S*,*S*)-Whelk-O1, MeOH–H_2O 75 : 25, 0.8 mL/min; detection at 280 nm): $\tau_{(S)}$ = 22.35 min, $\tau_{(R)}$ = 28.98 min

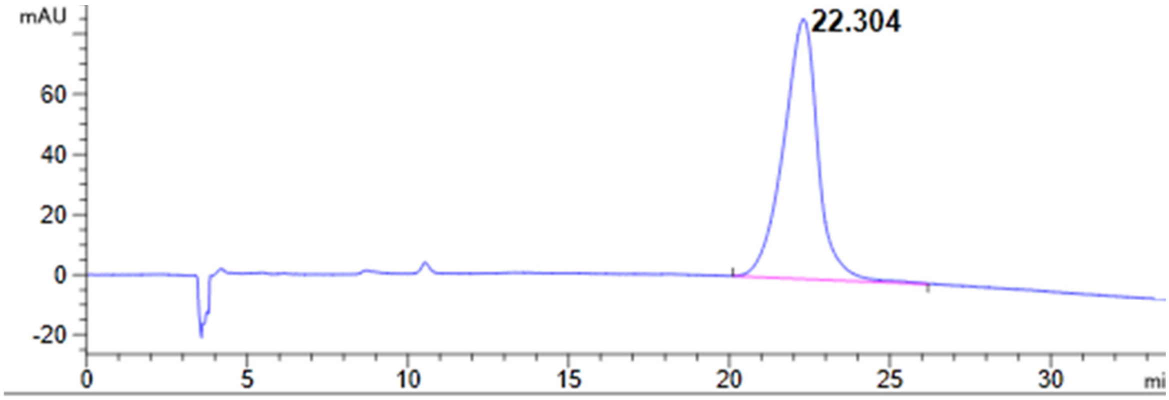

Peak #	RetTime [min]	Type	Width [min]	Area [mAU*s]	Height [mAU]	Area %
1	22.304	BB	1.0784	6463.74463	86.24223	100.0000

HPLC of compound (*S*)-**5e** ((*S*,*S*)-Whelk-O1, MeOH–H_2O 75 : 25, 0.8 mL/min; detection at 280 nm): $\tau_{(S)}$ = 22.30 min (> 99% *ee*)
